# A Highly Pure Sub-Fraction of Shallot Extract With Potent *in vitro* Anti-Angiogenic Activity

**Published:** 2014

**Authors:** Shima Famil Samavati, Hamid-Reza Mohammadi-Motlagh, Ali Mostafaie

**Affiliations:** 1 *Department of Biology, Islamic Azad University, Science and Research Branch, Tehran, Iran.*; 2 *Medical Biology Research Center, Kermanshah University of Medical Sciences, Kermanshah, Iran.*

**Keywords:** *Allium hirtifolium*, angiogenesis, phytochemicals, cancer, high performance thin layer chrom-atography

## Abstract

Our previous studies showed that various extracts of Persian shallot (*Allium hirtifolium*) have anti- angiogenic effects. This study has been undertaken to isolate and identify the major effective anti- angiogeneic sub-fraction of shallot. After preparation of the 50% hydroalcoholic extract of shallot bulbs, the extract was successively fractionated into n- hexane, ethyl acetate, n- butanol and aqueous fractions. Anti-angiogenesis activity of fractions was examined by *in* *vitro* angiogenesis assay. The ethyl acetate fraction which had the most anti-angiogenesis activity was further fractionated to four sub- fractions by thin layer chromatography (TLC), silica gel column chromatography and then analyzed by High Performance TLC (HPTLC) with ethyl acetate-methanol- water as the solvent system. Our results showed that one of the four sub- fractions, as the major band in HPTLC, had the most anti- angiogenic activity. Purification and characterization of the major anti- angiogenic compound/compounds of shallot's extract may constitute one means by which diets rich in shallot confer protection against cancer and finally introduce new agents with pharmacological activities in shallot as a potential candidate in cancer therapy.

Angiogenesis is a principle process of making new vessel formation from original ones. It can be found in adults during wound healing and also occurring in the female reproductive system (1). Any disturbance in angiogenesis process may result in the different pathological statuses (2). For example, rheumatoid arthritis (3), diabetic retinopathy (4), psoriasis and juvenile hemangio-mas (5), and finally, tumor growth and metastasis are due to the angiogenic imbalances (6). On the other hand, the deregulated angiogenesis is associated with disease progression especially tumor development. Thus, inhibition of angiogenesis is important in cancer prevention and also has recently become a target in drug development. Many studies have shown that the natural compounds of medicinal plants have diverse pharmacological activities and also advantages over synthetic drugs, such as moderate action and better tolerance (7). For example, plant derivatives such as genistein (8), isoliquitrin (9), ginsenoside (10) and torilin (11) have potent effects on endothelial cell proliferation and or tube formation. *Allium,* a genus of more than 500 species belongs to the family of *Liliaceae*. However, only a few of them are important as food plants, notably onion, garlic, shallot, chive, leek, and rakkyo. Such plants have been used for many centuries for their pungency and flavoring value, for their medicinal properties, and in some parts of the world, their use also has religious connotations (12-16). To date, there are few clinical reports about the pharmacological properties of shallot. These studies include: *in vitro* analysis of antioxidant activities of shallot extracts (17), confirmation of the the presence of flavone and polyphenolic derivatives such as quercetin, quercetin 4ˊ-glucoside, quercetin 7-glucoside, quercetin 4ˊ,3-diglucoside, and quercetin mono-D-glucose at high concentrations (18). Previously, we indicated that the aqueous extract of shallot bulbs has noticeable anti-angiogenic activity on *in* *vitro*, *in vivo* and *ex vivo* models without toxic effect on endothelial cells (19, 20). Besides, in an earlier study, we found that the aqueous extract of shallot inhibits the growth of several tumor cell lines and decreased the acute inflammation in mice models (21, 22). Furthermore, we reported that a flavonoid-rich fraction was responsible for the anti-angiogenic properties of shallot. The present study was done to partition the ethyl acetate fraction of shallot and reveal the responsible sub-fraction that abolishes angiogenesis *in vitro*.

## Materials and Methods

**Plant material**

Shallot bulbs (*A. hirtifolium L*.) were purcha-sed from the local vegetable market at Kermanshah (Iran) and verified at the Agricultural College of Razi University by Dr S. Maassoumi.

**Preparation of hydroalcoholic extract and subsequent fractions from fresh and dry shallot bulbs**

The preparation of hydroalcoholic extracts and subsequent fractions were performed using the successive fractionation as previously explained (23, 24). Firstly, the extracts were prepared by grinding and blending the shallot bulbs in a blender. The homogenates were extracted with 50% (v/v) ethanol by stirring for 24 h at 4 °C. The hydro-alcoholic extracts were filtered and centrifuged at 12,000×g for 20 min at 4ºC and then evaporated under reduced pressure to dryness. For solvent fractionation, the extracts were resuspended in distilled water, and then partitioned successively with n-hexane (Hex), ethyl acetate (EA) and n-butanol (BuOH), leaving a residual aqueous fraction (Aq). Each fraction was evaporated under reduced pressure to yield Hex, EA, BuOH and Aq fractions, respectively.

**Total flavonoids determination**

The determination of flavonoids was done with aluminum chloride colorimetric method as previously described (25). Briefly, each fraction (0.5 ml) was mixed with 1.5 ml of methanol, 0.1 ml of potassium acetate (1 M), 0.1 ml of aluminum chloride (10% w/v) and 2.8 ml distilled water. The mixtures remained at room temperature for 30 min, and then their absorbance was measured at 415 nm. The calibration curve was prepared using pure quercetin (Sigma Chemical Co., USA) solutions (as a standard flavonoid) at concentrations 15.62–125 µg/ml.

**Preparative thin layer chromatography (TLC)**

Ethyl acetate fraction was applied to aluminum-backed TLC plates coated with 0.2 mm layers of silica gel 60 F_254 _(Merck, Darmstadt, Germany). Pure quercetin was used as a control for comparison. The fraction and quercetin were spotted and placed in TLC tank. The mobile phase system was ethyl acetate–methanol–water (6:1:1.5). The plates were finally air dried and observed under UV light (254 and 366 nm) and R_f_values were calculated.

**Silica gel column chromatography**

Ethyl acetate fraction was applied to column chromatography Silica gel 60 F_254_. At first the column was washed with pure ethyl acetate solvent, then 1200 mg of the ethyl acetate fraction was loaded on the column. The mobile phase systems were ethyl acetate (100), ethyl acetate-methanol (95:5), ethyl acetate–methanol (90:10), and methanol –water (90:10).

**Cell culture**

Human umbilical vein endothelial cells (HUVECs) were obtained from the National Cell Bank, Pasteur Institute of Iran. Human umbilical vein endothelial cells (HUVECs) were taken out from the nitrogen tank and after melting at 37ºC, the culture medium was added and the mixture was centrifuged for 5 min at 1000 ×g. The pellet was washed for an additional time. After cell count and determining cell viability, the cells were re-suspended in DMEM: F12 medium (Gibco, NY, USA) containing 10% heat inactivated fetal bovine serum (FBS) (Gibco, NY, USA), 100 U/ml penicillin and 100 µg/ml streptomycin and trans-ferred into 25 cm^2^ cell culture flasks and maintai-ned at 37^o^C, 5% co2 in humidified incubator.

**HUVEC capillary tube formation in three-dimensional collagen gel.**

**Preparation of cytodex-3-microcarrier beads**

The cytodex-3-microcarrier beads (Amersham Pharmacia Biotech, USA) were pre-swelled in phosphate buffer saline (PBS), and then rinsed with DMEM: F12 medium under the class 2 laminar hood (26) and maintained at 4°C.

**Designing the three dimensional** ***in vitro*** **angiog-enesis model**

HUVECs at 90% confluency were trypsinized and mixed with cytodex-3-microcarriers at an appropriate ratio in DMEM: F12 medium supplemented with 10% heat inactivated fetal bovine serum (FBS), 100 U/ml penicillin and 100 µg/ml streptomycin. The mixing was longed 4 h and allowed for an additional incubation at 37 ºC incubator with 5% CO_2_ overnight. Then, the cell-coated beads were cultured in collagen matrix in a 24 well plate. In order to monitor the anti-angio-genesis effect of shallot fractions and quercetin, the wells were treated with different concentrations of fractions against controls, and the results were analyzed microscopically after 72 h using a specialized software package (AE-31; Motic) according to a standard method (27).

**High Performance Thin Layer Chromatography (HPTLC)**

Chromatography was performed on 20 cm×10 cm glass HPTLC plates precoated with layers of silica gel 60 F_254_ (E. Merck, Darmstadt, Germany) by a CAMAG (Muttenz, Switzerland) ADC2 Automatic TLC Sampler controlled by Win- CATS software. Different volumes (2.5, 5 and 10 µl) of sub- fraction solution at 1 mg/ mL were laid down. The plates were developed and observed to a Reprostar 3 illumination unit. The peak areas were recorded.

## Results

**Comparison of fractionation yield**

Based on the extraction step, each kg of the fresh shallot yielded 200g hydroalcoholic extract (20% w/w). The mentioned hydroalcoholic extract after fractionation was yielded n-hexane (0.57%), ethyl acetate (0.20%), n-butanol (6.20%) and aqueous (89.17%) fractions. In comparison, each kg of dry shallot yielded 700 g hydroalcoholic extract (70% w/w). The obtained fractions from the hydroalcoholic extract of dry shallot bulbs were n-hexane (0.65%), ethyl acetate (0.25%), n-butanol (7.15%) and aqueous (90.34%) fractions. Because of higher efficiency of the dry shallot bulbs, all the subsequent experiments were performed using dry shallot bulbs.

**Flavonoid contents of Hydroalcoholic extract and its fractions**

Flavonoid content of each fraction based onquercetin calibration curve (y = 0.005x + 0.010, r^2^ = 0.999) has been shown in Table 1. Ethyl acetate fraction had the highest flavonoid content (38% of total flavonoid) comparing with other fractions as the following order: Hex> BuOH> Aq.Table 1.

**Separation of ethyl acetate sub-fractions by TLC**

Quercetin was identified by observing the characteristic yellow zone. The R_f_ of quercetin was determined 0.9 based on the TLC experiment at 365 nm. The ethyl acetate fraction was separated to four sub-fractions (namely A, B, C and D). These sub-fractions appeared as four dark zones against a green background when exposed to UV light (254 nm). Furthermore, their R_f_ values were estimated as: A: 0.09, B: 0.45, C: 0.54 and D: 0.8.

**Inhibition of angiogenesis** ***in vitro*** **by shallot fractions**

The three-dimensional culture of HUVECs model was used to screen the inhibitory effects of the isolated fractions (EA, Hex, BuOH and Aq) obtained from the hydroalcoholic extract of shallot. After 72 h of treatment, untreated control wells showed a branching pattern of tube-like structures.

**Fig. 1:**
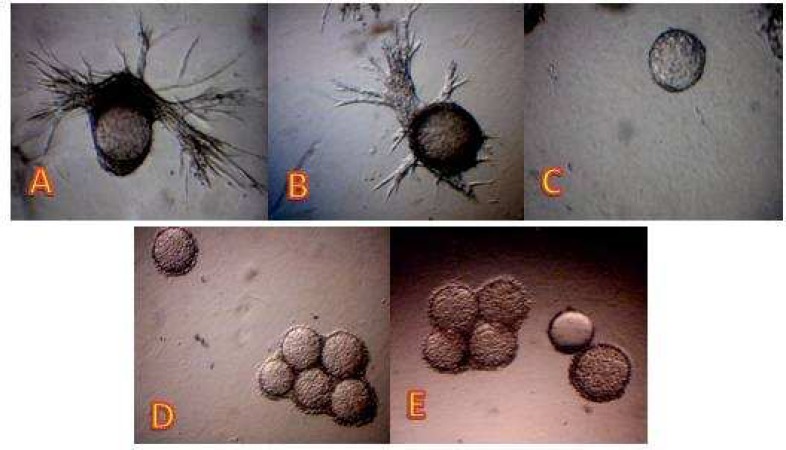
Effect of the EA fraction on *in vitro* angiogenesis model. Sprouting at control was induced by adding complete culture medium containing 10% FCS (A). Tube formation by endothelial cells was treated with EA fraction at 200 (B), 400 (C), 600 (D), 800 (E) and 1000 ng/ml (F).

Among the shallot fractions, the EA fraction had the most anti-angiogenic potential on three-dimensional *in vitro* model and this inhibitory effect was concentration-dependent. On the other hand, this fraction at concentrations up to 200 ng/ml could not inhibit the beads from sprouting tangibly. While with increasing concentration (400 ng/ml), the EA fraction caused significant inhibition of tube formation, and at 800– 1000 ng/ml inhibited angiogenesis completely (Fig. 1).

Our observations also showed that Hex fraction did not induce a significant decrease in the sprouting formation and progression by HUVEC at concentrations 200- 400 ng/ml but at 800 to 1000 ng/ml, it showed a significant inhibition. However, the 100% inhibitory effect was not obtained even at higher concentrations.

Interestingly, the BuOH and Aq fractions were not able to inhibit HUVEC tube formation at the used concentrations (200- 1000 ng/ml). These findings indicated that the BuOH and Aq fractions probably lack the responsible effective components in angiogenesis inhibition.

**Fig. 2:**
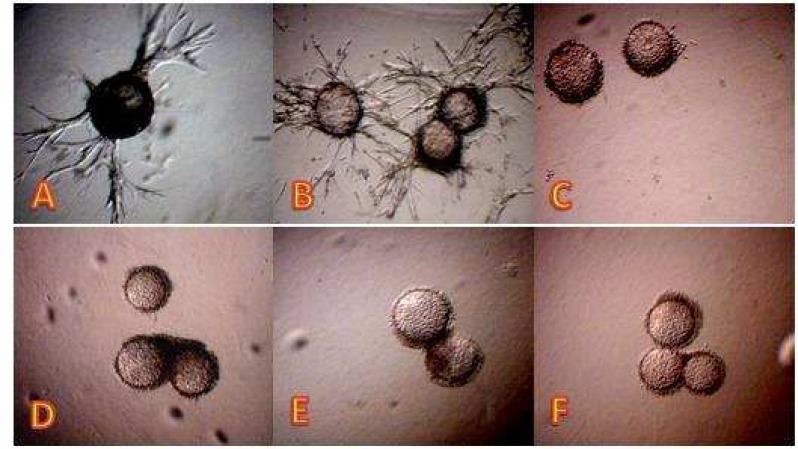
Anti-angiogenic effect of the pure quercetin flavonoid on *in vitro* angiogenesis model. Panel (A) is control. The wells were treated with quercetin at 5 (B), 10 (C), 25 (D), and 50 µg/ml (E).

In case of quercetin as a standard flavonoid, our results showed that it had no significant inhibitory effect on *in vitro* tube formation at concentrations 5 and 10 µg/ml. However, at 25 and 50 µg/ml of quercetin, complete inhibitory effect was observed (Fig. 2).

**Sub-fractions B and C had the most anti-angiogenic effects** ***in vitro***

**Fig. 3:**
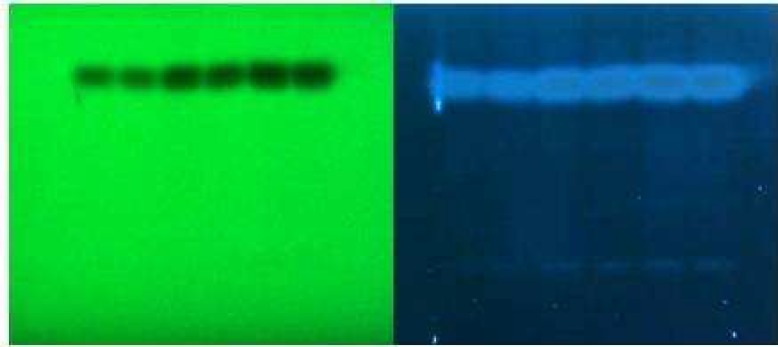
Anti-angiogenic effect of the sub-fractions (A to D) isolated from EA fraction by column chromatography. Panel (A) is the control at presence of complete culture medium without treatments. 100% inhibitory effect on tube formation was obtained by sub-fraction A at 800 ng/ml (B), sub-fraction B at 200 ng/ml (C), sub-fraction C at 200 ng/ml (D), and sub-fraction D at 800 ng/ml (E).

Among the EA sub-fractions, the sub-fraction A at 1000 ng/ml, sub-fractions B and C at 200 ng/ml, and D at 800 ng/ml could completely inhibit tube formation in three-dimensional collagen-cytodex model (Fig. 3).

**Purity examination of sub-fraction B by HPTLC**

**Fig. 4:**
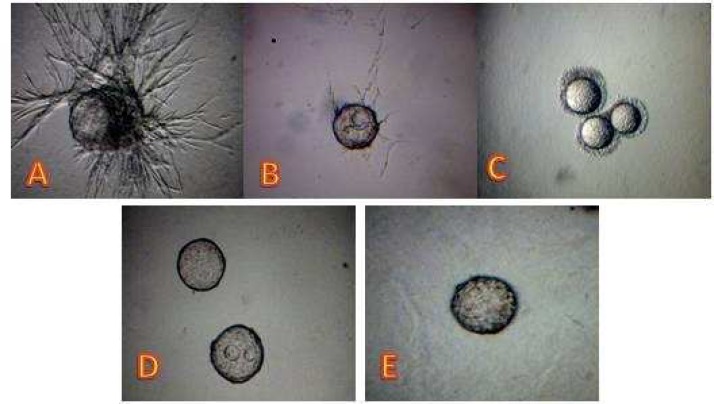
HPTLC analysis of sub-fraction B in 254 nm (Right) and 366 nm (Left) at different duplicated loaded volumes (2.5, 5 and 7.5 µl per spot).

With the modification of our isolation method in silica gel column chromatography, we realized that the sub-fraction C contained both sub-fraction B and C and in fact its anti-angiogenic effect was due to the presence of sub-fraction B. Thus, HPTLC as an analytical method was used for the purity identification of sub-fraction B as the potential candidate responsible in angiogenesis inhibition. Together with the findings obtained by the TLC method, the results of HPTLC analysis also indicated a high purity (at least 98%) of sub-fraction B at 254 and 366 nm (Fig. 4).

**Table 1:** Flavonoid quantities in the fractions of shallot

Sample	Volume of sample (ml)	Concentration of Flavonoid (µg/ml)	Amount of flavonoid (µg)	flavonoid in sample per hydroalcoholic extract* (%)
Hydroalcoholic extract	20	50	525	100
Hex fraction	2	1	60.14	11.45
EA fraction	3	1.66	182.25	34.71
BuO fraction	8	8.75	82.4	15.69
Aq fraction	4	187.5	160.44	30.56

* Each value was obtained by calculating the average of at least three experiments.

## Discussion

In continuing with our previous studies on anti-angiogenic activity of Persian shallot, in the present research we showed that some fractions from shallot are effective inhibitors of angiogenesis *in vitro*. Recent investigations have shown that *Allium *species and their constituents have anti-cancer effects (28). These effects are supported by epidemiological data from population based studies (29, 30). Although numerous scientific studies have been undertaken on other members of *Allium* family such as garlic to survey the basis and validity of these beliefs, a few studies have been carried out on shallot (31). In this study, anti-angiogenic activity of all fractions was carried out on *in vitro* endothelial cells-coated cytodex in 3D collagen matrix to find the fraction with the highest activity. Our results from the anti-angiogenic potential of ethyl acetate fraction showed that this fraction could inhibit angiogenesis completely from 400 ng/ml. On the other hand, EA fractions in a dose-dependent manner had potent inhibition of sprouting and capillary tube formation on HUVEC, without considerable toxic effect on the cells even up to 2000 ng/ml. In 1992, Leighton et al. (32) reported that shallot contains the highest level of total flavonols among the onion varieties. Furthermore, the findings of Fattorusso et al. in 2002 showed that bulbs of shallot have high concentrations of quercetin, isorhamnetin, and along with their glycosides forms (18). Furthermore, Yang et al. in 2004 (33) also reported that acetone extract of shallot has high flavonoid content. They used catechin as a standard flavonoid. We also found that the hydroalcoholic extract of shallot is rich in total flavonoid (quercetin was used as the standard flavonoid). Besides, the ethyl acetate fraction of shallot, which exhibited the greatest anti-angiogenic activity, contained the highest amount of flavonoid compounds, among the other fractions including Hex, BuOH and Aq (Table1). 

Quercetin is an important constituent of the flavonoid family and is already found in high concentrations in shallot. The anti-angiogenic activity of quercetin was also previously documented (34, 35). Herein, we found that quercetin (as positive control) inhibited *in vitro* angiogenesis at microgram per milliliter (µg/ml) concentrations compared to the nanogram (ng/ml) concentrations of ethyl acetate fraction and its major sub-fractions. On the basis of the study by Tan et al. (2003), quercetin as a standard flavonoid inhibits several important steps of angiogenesis including proliferation, migration, and tube formation of endothelial cells *in vitro* and all these effects are concentration-dependent (34). In a more recent study, we have observed that shallot extract has considerable inhibitory effect on migration and proliferation of the endothelial cells. Our results also showed that the extract down-regulates vascular endothelial growth factor (VEGF), and also matrix metalloproteinases (MMP-2 and MMP-9) at the mRNA and protein levels. These factors are as major factors in initiation and progression of the angiogenic proccss that is associated with tumor growth and metastasis in several human malignancies (36-38). However, our findings (39, 40) have confirmed the recently published findings of Tan et al., but as mentioned above, on the basis of the results of anti-angiogenic assay, there is a great difference between the effective concentrations of the quercetin (µg/ml) and the effective concentration of ethyl acetate fraction and its sub-fractions (ng/ml). Thus, it may be concluded that contrary to what is expected, it is unlikely that the flavonoids compounds present in shallot are the major factors responsible of the anti-angiogenic activity.

In addition to polyphenolic phytochemical derivatives, some studies have been reported that Persian shallot contains abundant amounts of sulfide ingredients. On the other hand, shallot like other *Allium *species contains organosulfur compounds including allicin-decomposition products (diallyl disulfide, diallyltrisulfide, and ajoene), S-allyl cysteine were shown to have potential antitumor activities. Because of abundance of organosulfur compounds of *Allium* plants, these compounds may be responsible for some beneficial properties of these plants. Thus, the biological properties of *Allium *plants such as anti-angiogenesis and anti-cancer effects could be more possibly attributable to organosulfur ingredients (41). Therefore, the active compounds in shallot which are responsible for anti- angiogenic activity may be one or combination of the aforesaid phytochemical compounds. Our results of preparative TLC indicated that EA fraction can be isolated to at least four sub-fractions. Among the isolated compounds, sub-fractions B and C showed significant anti-angiogenic activities. The result of analytical HPTLC also demonstrated the purity of sub-fraction B by 98%. These findings also help us in identifying the structure. Further investigations should be conducted to isolate and characterize the responsible compound/compounds by analysis methods and also to clarify its/their possible mechanisms in this regard.

In conclusion, this study has provided evidence of anti-angiogenesis activity of responsible constituents of the fractions and its sub-fractions isolated from shallot bulbs* in vitro*. These findings emphasis on to improve current dietary advice by actively promoting increased consumption of shallot plant as an essential means to prevent cancer or even to treat cancer. Although exploration of the molecular mechanisms on anti-angiogenesis activity of active compounds from shallot need further research.
